# Defining Swelling Kinetics in Block Copolymer Thin Films: The Critical Role of Temperature and Vapour Pressure Ramp

**DOI:** 10.3390/polym13234238

**Published:** 2021-12-03

**Authors:** Sudhakara Naidu Neppalli, Timothy W. Collins, Zahra Gholamvand, Cian Cummins, Michael A. Morris, Parvaneh Mokarian-Tabari

**Affiliations:** 1 School of Chemistry, The University of Dublin, Trinity College Dublin, D02 PN40 Dublin, Ireland; naidunes@tcd.ie (S.N.N.); gholamvz@tcd.ie (Z.G.); morrism2@tcd.ie (M.A.M.); 2 Advance Material and BioEngineering Research (AMBER) Centre and CRANN, Trinity College Dublin, D02 PN40 Dublin, Ireland; 3 Department of Chemistry, University College Cork, Tyndall National Institute, T12 K8AF Cork, Ireland; collins.incorporated@gmail.com; 4 Centre de Recherche Paul Pascal (CRPP), The French National Centre for Scientific Research (CNRS), University of Bordeaux, UMR 5031, 115 Avenue Schweitzer, 33600 Pessac, France; cian.a.cummins@gmail.com; 5 Laboratoire de Chimie des Polymeres Organiques (LCPO), University of Bordeaux, CNRS, Bordeaux INP, 16 Avenue Pey-Berland, CEDEX, 33607 Pessac, France

**Keywords:** block copolymer, phase separation, solvent vapour annealing, ramp rate, kinetic mechanism

## Abstract

We studied the kinetics of swelling in high-χ lamellar-forming poly(styrene)-*block*- poly(lactic acid) (PS-*b*-PLA) block copolymer (BCP) by varying the heating rate and monitoring the solvent vapour pressure and the substrate temperature in situ during solvo-thermal vapour annealing (STVA) in an oven, and analysing the resulting morphology. Our results demonstrate that there is not only a solvent vapour pressure threshold (120 kPa), but also that the rate of reaching this pressure threshold has a significant effect on the microphase separation and the resulting morphologies. To study the heating rate effect, identical films were annealed in a tetrahydrofuran (THF) vapour environment under three different ramp regimes, low $(r_{T}<1\;{}^{\circ}C/\min$), medium $\left(2<r_{T}<3\;{}^{\circ}C/\min\right)$ and high $\left(r_{T}>4\;{}^{\circ}C/\min\right)$, for 60, 90 and 120 min, respectively, while the solvent vapour pressure and the substrate temperature were measured in real time. The translational order improved significantly with increasing the heating rate. The solvent mass uptake calculated for the different ramp regimes during annealing is linearly proportional to time, indicating that the swelling kinetics followed Case II diffusion. Two stages of the swelling behaviour were observed: (i) diffusion at the initial stages of swelling and (ii) stress relaxation, controlled at later stages. Films with a faster rate of increase in vapour pressure $(r_{P}>2\;\mathrm{kPa}/\min$) reached the pressure threshold value at an early stage of the swelling and attained a good phase separation. According to our results, highly ordered patterns are only obtained when the volume fraction of the solvent exceeds the polymer volume fraction, i.e., $\left(\varphi_{s}\geq \varphi_{p}\right)$*_,_* during the swelling process, and below this threshold value $\left(\varphi_{s}=0.5\right)$, the films did not obtain a good structural order, even at longer annealing times.

## 1. Introduction

Solvent vapour annealing (SVA) and solvo-thermal vapour annealing (STVA) are alternatives to the thermal annealing of block copolymers (BCPs) that avoid the issues of thermal degradation, slow chain kinetics and the corresponding extended heating times [1,2,3]. In the SVA approach, the films are exposed to solvent or mixed solvent vapours that can be preferential or non-preferential for a particular block. The uptake of these solvents induces swelling, plasticisation and chain movement, thereby enhancing the microphase separation and the formation of long-range ordered BCP patterns. In recent years, the SVA and STVA of BCP thin films have been extensively used to obtain the long-range order, lyotropic transitions and the morphology selection [4,5,6,7,8,9,10]. Long-range ordered BCP thin films have potential in many applications, including nano lithography [11,12,13,14], optoelectronics [15,16], optics [17], sensors [18], storage devices [19] and membranes [20,21]. To exert control of the BCP microphase separation, researchers have extensively investigated swelling and the related process parameters during SVA, including the solvent selection [22,23,24,25], vapour pressure [26], temperature [27,28], swelling ratio [24,25,29,30], morphology [31], thickness [32], solvent evaporation rate [8,33,34] and orientation [33,35].

Although swelling and the related in situ studies of polymers have received considerable attention for several decades [36,37,38,39,40,41,42,43,44,45,46,47,48,49,50,51,52,53,54], studies of BCP swelling kinetics during solvent exposure are less common [4,5,6,33,34,55,56]. Lundy et al. [57] studied how the annealing conditions (annealing time, bubbler temperature, solvent vapour saturation) affect the phase segregation kinetics of poly(styrene)-*block*-poly(4-vinylpyridine) (PS-*b*-P4VP) films, enabling the minimising of the annealing times while maintaining pattern fidelity over large areas. Selkirk et al. [56] investigated and optimised the self-assembly kinetics of a large poly(styrene)-*block*-poly(2-vinylpyridine) (PS-*b*-P2VP) system, examining the effects of both the rate of the swelling and the hold time on the lateral ordering. Phillip et al. [33] reported that the rate of nucleation and growth of the cylindrical domains depends on the solvent concentration in the swollen films and the solvent evaporation rate on the orientation of PS-*b*-PLA BCP. The role of the specific SVA pathway on the swelling ratio, morphology, orientation and grain size were elucidated by Jung et al. [24] by following different processing protocols while annealing the poly(styrene)-*block*-poly(dimethylsiloxane) (PS-*b*-PDMS) films in a mixture of solvents. Kreuzer et al. [58] investigated the swelling and the exchange kinetics by exposing poly(sulfobetaine)-based BCP thin films to H_2_O (water) and D_2_O (deuterated) vapours, and showed the effect of the polymer–solvent interactions on the swelling behaviour of the films. The influence of the substrate on the swelling kinetics, steady-state solvent uptake and chain dynamics was reported by Ogieglo et al. [5] and Stenbock et al. [59]. Polystyrene-*b*-polybutadiene (PS-*b*-PB) thin films on polymer network (PN) supports and silicon substrates were subjected to stepwise increases of the partial vapour pressure of chloroform and showed the influence of the substrate on the swelling of the films. In this work, we study how the different kinetic pathways affect the self-assembly of poly(styrene)-*block*-poly(lactic acid) (PS-*b*-PLA) BCP during the STVA process in a conventional oven. Specifically, we explore the effect of heating and vapour pressure ramps on the swelling of the BCP thin films. The swelling process in polymers is a combination of the diffusion of the solvent to the free volume present in the porous structure of the polymer (mass uptake) and the local relaxation of the polymer chains. In general, the mass transport is described in the framework of Fick’s laws. The kind of diffusion in which the mass of the diffusive species is determined as a function of time, and which is proportional to the square root of time, is called Case I diffusion. This is distinguished from Case II diffusion, in which the mass uptake changes linearly with time. Case II diffusion [60,61] is characterised by linear kinetics and a sharp diffusion front, and can be described in two main stages: (a) the initiation stage, in which the volume fraction of the solvent on the surface will increase until a critical value is reached, at which point a Case II diffusion front forms; and (b) the propagation stage, where the front moves through the polymer with a constant speed. The polymer ahead of the front is glassy but the material behind it is highly plasticised. In Case II diffusion, which usually takes place in glassy polymers, the relaxation time is much longer than the time associated with diffusion.

Although there are numerous studies on the usage of solvent annealing for microphase separation in films, both in conventional and environmentally controlled apparatus [4,57,62,63], the role of annealing parameters such as vapour pressure and temperature ramp on swelling kinetics is not well understood. Cheng et al. [4] studied the vapour pressure effect on the solvent concentration in swollen films by the SVA of PS-*b*-PLA films in toluene and chloroform at a constant temperature. The greater vapour pressure of chloroform to toluene at a constant temperature leads to more solvent uptake in the swollen films. They also found that the temperature of the solvent vapour has a significant effect on the solvent volume fraction in the films at a constant substrate temperature. Baruth et al. [64,65] reported four solvent regimes in the SVA process conducted in a custom-made ‘rig’ apparatus: (i) initial solvent uptake, (ii) metered solvent uptake, (iii) fixed solvent concentration and (iv) solvent evaporation. The swelling rate in the metered solvent uptake regime has no obvious impact on the final morphology of the PS-*b*-PLA film; however, a vapour pressure effect on the correlation length was observed. Jin et al. [66] designed a feedback control SVA apparatus using gas flow mixing to precisely control the degree of the swelling, the swelling rate and the evaporation rate of the solvent. Recently, Hulkkonen et al. [63] also designed an automated SVA system which uses local heating or cooling to control the swelling behaviour and that is more effective and faster than the gas mixing. They also studied the effect of the swelling ratio and the swelling time on the morphology and the lateral order of the PS-*b*-P4VP films, respectively.

## 2. Experimental Section

### 2.1. Materials and Methods

Lamellar-forming poly(styrene)-*block*-poly(lactic acid) (PS-*b*-PLA) BCP was purchased from Polymer Source (Dorval, Canada) with the number average molecular weight of *M_n,PS_* = 21 kg mol^−1^ and *M_n,PLA_* = 19.5 kg mol^−1^, and the volume fraction of *f_PS_* = 0.55 with PDI of 1.06, and was used as received. All solvents used in this study were HPLC grade (≥99.9%) and purchased from Sigma-Aldrich (Wicklow, Ireland). Chloroform was used as a solvent for polymer dissolution while tetrahydrofuran (THF) was employed for solvo-thermal vapour annealing. Toluene and isopropanol alcohol (IPA) were used for cleaning. The 2 × 2 cm^2^ silicon substrates were cleaned by sonication in toluene, followed by IPA for 15 min each and then dried under a stream of nitrogen. Afterwards, the substrates were exposed to UV/ozone for 30 min to remove residual organic contaminant prior to spin-casting. Thin films of PS-*b*-PLA were spun cast from a 2 wt.% chloroform solution onto the cleaned silicon substrates to yield a film thickness of 217 ± 3 nm.

### 2.2. Experimental In Situ Set-Up: Monitoring Substrate Temperature and Solvent Pressure during Solvo-Thermal Vapour Annealing (STVA)

STVA was performed in an air-tight borosilicate bell jar (100 mL) containing 3 mL of THF solvent, connected to a pressure gauge for in situ monitoring of the pressure (see Figure S1 in ESI). The values of absolute vapour pressure were collected and recorded at 0.02 s intervals. Annealing jars were placed on different thermally conducting materials, which were then placed in the oven to create the various heating ramp regimes. An external type K thermocouple was attached to the silicon substrate and to a sensor (PASPort TM) in order to directly measure the temperature of the substrate during the annealing process (see Figure S1). The target oven temperature was set at 55 °C [67], and samples were annealed for 60, 90 and 120 min. The correlation profiles of absolute pressure versus temperature and pressure ramp versus heating ramp, annealed for 60 min time intervals, are shown in Figure S3. The ramp regimes were determined by applying the appropriate linear fittings to the measured temperature–time and pressure–time plots (Figures 2 and 3). The ramp regimes are listed in Table 1. After the desired annealing time, the samples were removed from the glass jars to allow the trapped solvent to evaporate at ambient temperature. It was observed that the solvent vapour-annealed samples were not visibly different from the as-cast films.

### 2.3. Solvo-Microwave Annealing

Although the focus of this paper is on the annealing process in a conventional oven, we have also used an industrial microwave unit (CEM) to create an ultrahigh pressure ramp (>7 kPa/min), greater than that achievable in an oven, for the purpose of comparison (1). To monitor the pressure of the solvent during solvo-microwave annealing, a custom-made microwave glass was fabricated in order to locate the pressure gauge on top of the vessel (Figure S2). A microwave power of 50 W was applied to reach the target temperature of 55 °C [27].

### 2.4. Film Characterisation

The surface topography of the annealed films was imaged using atomic force microscopy (AFM) (Park Systems, XE-7, Trinity College, Dublin, Ireland) operated in AC (tapping) mode under ambient conditions using silicon microcantilever probe tips with a force constant of 42 N m^−1^. Each scan was run over a 2 μm × 2 μm area, and each sample was scanned at multiple areas over the surface to determine the consistency of phase separation. Topographic images were analysed using XEI image processing and Analysis software from Park Systems.

## 3. Results

### 3.1. Ramp Effect on Phase Separation

In our previously published work on solvo-microwave annealing [27], we highlighted the fast heating rate of the polar solvents in the microwave, which led to self-assembly in the PS-*b*-PLA film in less than 1 min. Applying the Antoine equation, ($P=10^{A-\frac{B}{C+T}}$), we argued that the nominal vapour pressure of the THF increases from 19.8 kPa at room temperature to 70 kPa at 55 °C within seconds of microwave exposure. The high vapour pressure of the THF in a sealed vessel provides a fast access for diffusion to the depth of the film, which gives the polymer chains enough mobility to microphase separate into well-ordered domains. However, we were not able to measure the vapour pressure in the sample due to the practical limitations within the microwave apparatus. In the present work, we have modified the microwave vessel to be able to monitor the vapour pressure of the THF during the annealing of PS-*b*-PLA films in the microwave and in the oven during STVA. This allowed us to comprehensively evaluate the significance of the pressure as well as the rate of the pressure applied on the PS-*b*-PLA nanopattern formation. Indeed, we hypothesised that by controlling and monitoring the heating rate and pressure ramp in situ during the oven and microwave annealing, we could unravel the kinetic pathways of the pattern formation. Note: The focus of the current article is not on microwave annealing; however, the purpose of presenting the microwave result in 1 is to provide the preliminary proof of concept for the significant effect of high vapour pressure on the self-assembly process of block copolymers, and to reach a high vapour pressure level that is otherwise not achievable in a conventional oven.

**Figure 1:**
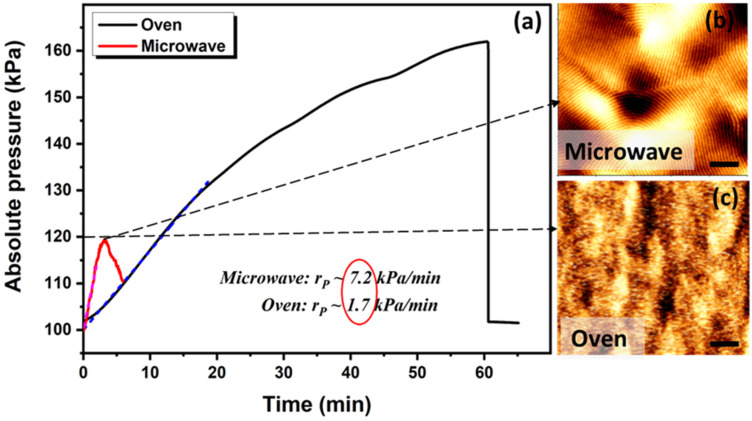
**The effect of osmotic pressure ramp on phase separation.** (**a**) In situ measurement of vapour pressure during annealing of PS-*b*-PLA film in conventional oven with lower ramp of $\left(r_{P}=1.7\;\mathrm{kPa}/\min\right)$, and in the microwave with the rapid pressure ramp of $\left(r_{P}=7.2\;\mathrm{kPa}/\min\right)$. Topographic AFM images of the SVA film in the microwave (**b**), and oven (**c**), at the point when the vapour pressure reached 120 kPa. Scale bars are 300 nm.

The in situ absolute pressure (*P_abs_*) was measured over time under different regimes in the conventional oven and in the microwave, as shown in Figure 1a. When the absolute pressure reached the threshold value of 120 kPa, the samples were removed from the annealing chambers and imaged by AFM (1b and 1c). We refer to 120 kPa as the threshold pressure; below this value, microphase separation was not observed. However, this experimental threshold value is based on our observations and results for this particular system and solvents, which may defer from other BCPs and processing conditions. Note that the absolute pressure measured is the sum of the atmospheric pressure (approximately 100 kPa) and the solvent vapour (THF) pressure. Figure 1 clearly demonstrates the significance of the pressure ramp on the long-range order of the PS-*b*-PLA film. While both films reached the same vapour pressure (120 kPa) during SVA, the film annealed in the microwave with a higher pressure ramp is segregated into perpendicular lamella of the PS-*b*-PLA (1b), whereas the film annealed in the oven with a lower pressure ramp is still in the disordered state (1c). Despite the longer annealing time (and the relatively similar vapour pressure), microphase separation did not occur for the sample treated in the oven. The major processing difference between the two films shown in Figure 1b,c is the rate of heating, which affects the rate of the osmotic pressure increase. The vapour pressure of the solvent during microwave annealing reached 120 kPa in the shorter time interval (<3 min) with a ramp $\left(r_{P}\right)$ of 7.2 kPa/min, whereas it took 12 min in the conventional oven with a ramp $\left(r_{P}\right)$ of 1.7 kPa/min to reach the same pressure. The pressure profiles and the AFM images in Figure 1 emphasise that, irrespective of the vapour pressure reaching its threshold value, the pressure ramp greatly influences the lateral order in the PS-*b*-PLA BCP films. To rule out any disparity arising from the different kinetic mechanisms in the microwave and the oven, we studied the effect of temperature and pressure ramp in the STVA samples annealed in the oven. This is due to the solvent molecules possessing vibrational energy in the oven, whereas in the microwave, the electronically and rotationally excited solvent molecules have high energy states besides thermal.

**Figure 2:**
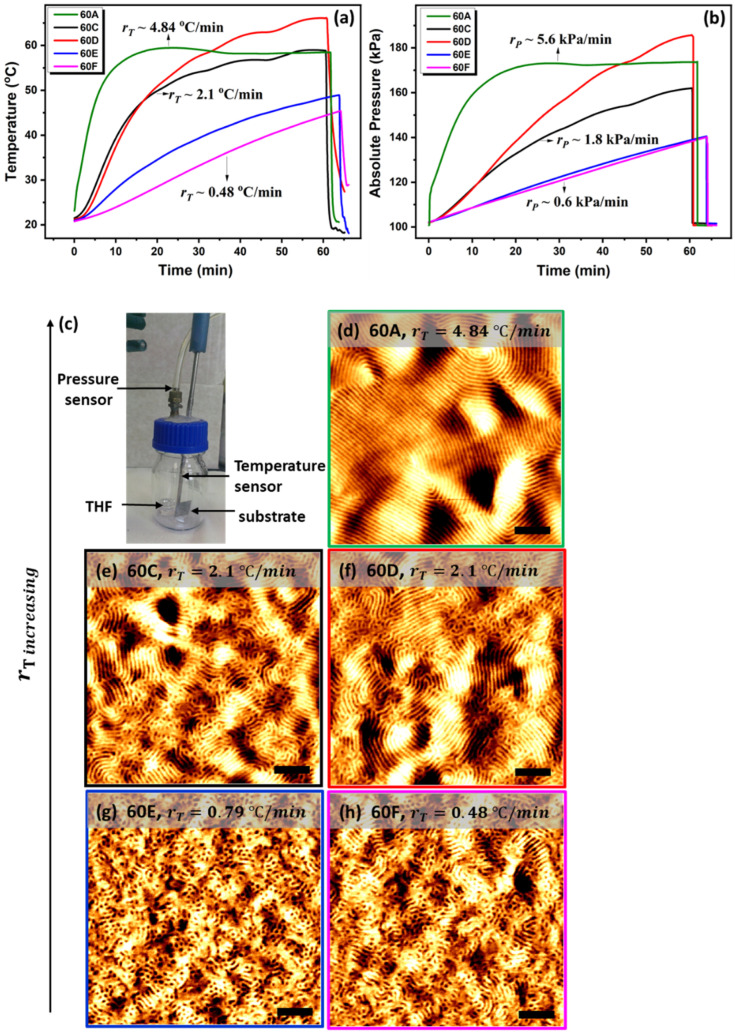
(**a**) Temperature and (**b**) absolute vapour pressure profiles for the films annealed in oven at different temperature ramps for 60 min. Higher temperature ramp leads to higher degree of order. AFM images (scale bars 300 nm) show high coherence length for (**d**) high heating ramp ($r_{T}\sim 4.84\;{}^{\circ}C/\min$, sample 60A); moderate phase separation for (**e**,**f**) medium heating ramp ($r_{T}\sim 2.1\;{}^{\circ}C/\min$, samples 60C and 60D); poor phase separation for (**g**,**h**) low heating ramp ($r_{T}\leq 0.76\;{}^{\circ}C/\min$, samples 60E and 60F). Image (**c**) shows the annealing jar with temperature and pressure sensors.

**Figure 3:**
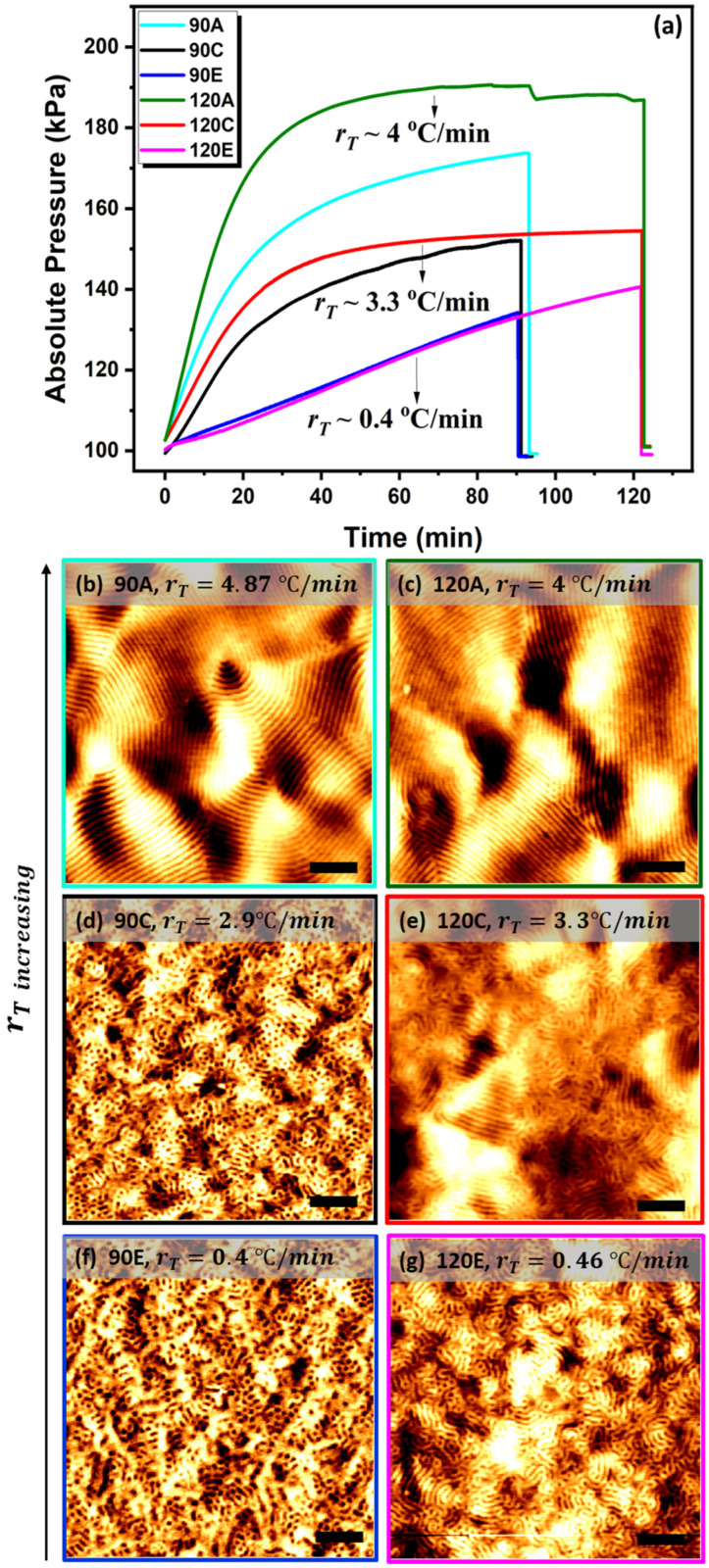
**The significance of pressure ramp over the length of the annealing time**. Absolute vapour pressure (**a**) and relevant AFM images (**b**–**g**) for the films annealed in oven at different ramps for 90 min (samples 90A (**b**), 90C (**d**), 90E (**f**)) and 120 min (samples 120A (**c**), 120C (**e**), 120E (**g**)). AFM scale bars are 300 nm. At low heating ramp, increasing the annealing time does not appear to improve the order.

The PS-*b*-PLA films were annealed in the oven with different heating ramps and slopes of temperature–time curves, with an indicative increase of temperature per minute, while exposed to THF for 60 min at 55 °C. The in situ measured absolute vapour pressure profiles at different temperature ramps are listed in Table 1, and the corresponding AFM images are shown in Figure 2. Although all the films reached the threshold vapour pressure (120 kPa), there is a distinctive difference in the degree of the order between the rapidly and slowly annealed films. The films (samples 60E and 60F, Figure 2) annealed with a lower temperature and pressure ramp ($r_{T}<1\;{}^{\circ}C/\min$ and $r_{P}<1\;\mathrm{kPa}/\min$) show a mesh-like structure with minimum line length. Increasing the ramp ($r_{T}>2.1\;{}^{\circ}C/\min$ and $r_{P}>2\;\mathrm{kPa}/\min$) induces better ordering rearrangement (samples 60C and 60D, Figure 2) with increased coherence length, and when the temperature ramp reaches above 4 °C/min ($r_{T}>4\;{}^{\circ}C/\min$ and $r_{P}>5\;\mathrm{kPa}/\min$), the films show a long-range ordered microphase-separated pattern (60A in Figure 2d). We noticed that, despite sample 60D (with medium $r_{T}\sim 2.1\;{}^{\circ}C/\min$) reaching a higher temperature than sample 60A (with higher $r_{T}\sim 4.84\;{}^{\circ}C/\min$), at a later stage of the annealing it shows only a moderate microphase separation in comparison to sample 60A, which imparts a higher degree of order. When the temperature for sample 60D was overshot to 66 °C, approaching the boiling point of THF (66 °C), the vapour pressure of the solvent abruptly increased to 185 kPa, higher than that of 60A. Attaining only moderate lateral order in sample 60D affirms the importance of the heating ramp overreaching the higher temperature or the vapour pressure. It also suggests that the rate of the heating and the osmotic pressure built up is more important at the early stage of the swelling than the later stage. This is potentially due to the higher osmatic pressure difference built up by the higher heating ramp, allowing for a larger amount of solvent to diffuse through the film at early stage of swelling, which overcomes the stress relaxation in the later stage to improve the translational order. We will discuss this in more detail in the swelling kinetics section. For the films heated with a ramp greater than 2 °C/min (Sample 60A, 60C, 60D, Figure 2), the absolute vapour pressure of the solvent was close to saturation (178 kPa at the target temperature of 55 °C) after 60 min. However, the films heated at a slower rate did not reach the same pressure. The films annealed at lower heating rates (sample 60E and 60F, in Figure 2) never reached the target temperature (55 °C) during the hour interval, thereby necessitating the longer annealing times of 90 and 120 min. Figure 3 shows the absolute pressure profiles at various temperature ramps for these extended annealing times and their corresponding AFM images (Figure 3b–h). The longer annealing times did not enhance the coherence length significantly, as is evident from Figure 3c,e,g. Although the heating rate for 90C (2.9 °C/min) in Figure 3d is higher than that of 60C (2.1 °C/min) and 60D (2.1 °C/min) in Figure 2d, we observed that the pressure ramp built up in the chamber for 90C (1.6 kPa/min) is lower than 60C (1.8 kPa/min) and 60D (2 kPa/min) due to the processing error results poor phase separation. These results affirm the importance of pressure ramp over temperature.

### 3.2. Swelling Kinetics

#### 3.2.1. Extraction of Polymer Volume Fraction

To further understand the observed pattern evolution, we examined the system’s associated polymer ($\varphi_{P}$) and the solvent’s volume fractions ($\varphi_{s}$), mass uptake (*M*) and swelling ratio ($d/d_{0}$), where $d_{0}$ is the initial dry film thickness and d is the thickness of the swelled film during the swelling process [64]. Instead of the common approach of extracting the parameters from the thickness measurement, we followed different approaches where there was no possibility to measure the thickness in situ during the swelling, for example, in the conventional oven. We monitored the absolute pressure of the solvent and the temperature of the film during the annealing. Subsequently, we employed Equation (1) to derive the relationship between the vapour pressure, the polymer volume fraction and the Flory–Huggins interaction parameter (*χ*) between the solvent and the polymer to extract the polymer volume fraction ($\varphi_{P}$) using Mathematica software.

(1) $$ln\left(\frac{P}{P_{0}}\right)=\chi_{SP}\varphi_{P}^{2}+ln\left(1-\varphi_{P}\right)+\left(1-\frac{1}{N}\right)\varphi_{P}$$

*P* is vapour pressure during swelling ($P=P_{abs}-100$) and *P*_0_ is the vapour pressure of the THF at a given temperature as determined by the Antoine equation ($P\left(T\right)=10^{A-\frac{B}{C+T}}$). $\chi_{SP}$ is the Flory–Huggins interaction parameter between the solvent and the polymer, derived from Equation (2),

(2) $$\chi_{SP}=f_{A}\;\chi_{SA}+\left(1-f_{A}\right)\chi_{SB}+f_{A}\;\left(1-f_{A}\right)\chi_{AB}\left(\frac{V_{S}}{V_{AB}}\right)$$

where $f_{A}$ is the volume fraction of the PS block ($f_{A}$ = 0.55), $\chi_{SA}$ is the interaction parameter between the solvent and PS ($\chi_{SA}$ = 0.15), $\chi_{SB}$ is the interaction parameter between the solvent and PLA ($\chi_{SB}$ = 0.62), $\chi_{AB}$ is the interaction parameter between PS and PLA ($\chi_{AB}=\left(98.1/T\right)-0.112$) and $V_{s}$ and $V_{AB}$ are the molar volumes of the solvent and the block copolymer, respectively.

#### 3.2.2. Solvent Volume Fraction on Phase Separation

**Figure 4:**
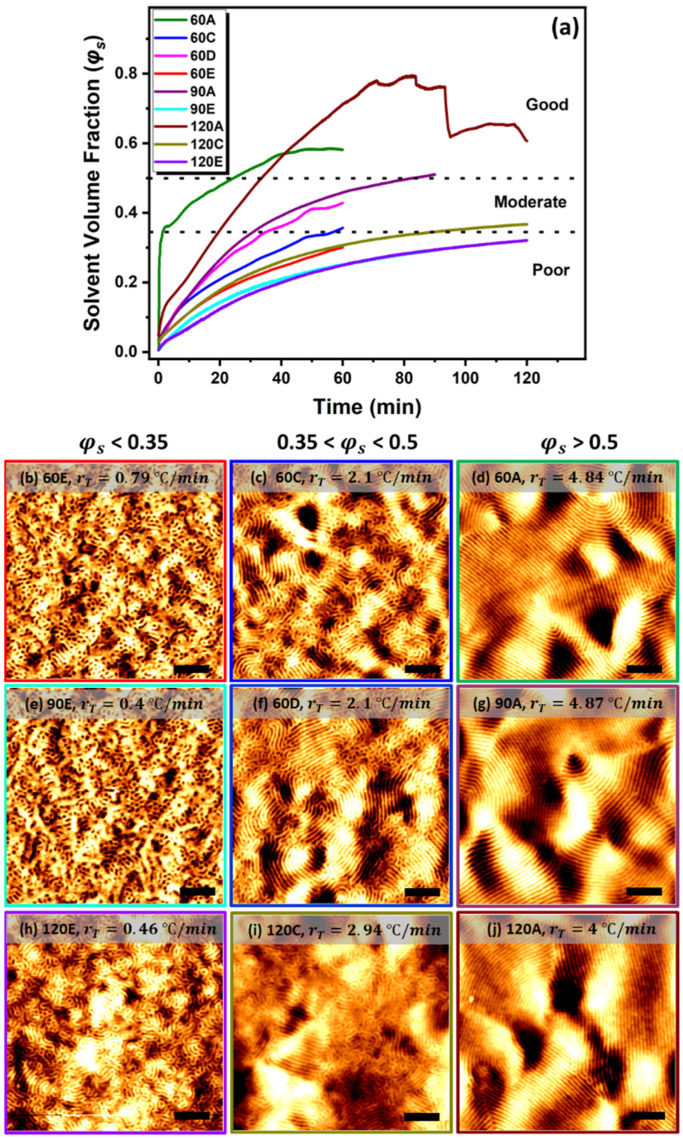
**The effect of heating ramp on solvent uptake.** (**a**) Evolution of solvent volume fraction ($\varphi_{s}$) in the swollen films during slow and fast heating regimes, and various annealing intervals. Corresponding AFM images show how the solvent volume fraction imposes different degrees of the lateral order in PS-*b*-PLA film (poor/moderate/good): (**b**,**e**,**h**) showing poor phase separation $\left(\varphi_{s}<0.35\right)$; (**c**,**f**,**i**) showing moderate phase separation $\left(0.35<\varphi_{s}<0.5\right)$; (**d**,**g**,**j**) showing good translational order $\left(\varphi_{s}\geq 0.5\right)$. The AFM scale bars are 300 nm.

The solvent volume fraction ($\varphi_{s}=1-\varphi_{p}$) in the swollen polymer is estimated from Equation (1) to evaluate the threshold solvent volume fraction to achieve a good microphase separation, as seen in Figure 4. The solvent volume fraction increases significantly with the heating ramp, i.e., the solvent diffusion rate strongly correlates with the heating ramp. In the films annealed with a lower ramp, $r_{T}<1\;{}^{\circ}C/\min$ (samples 60E, 90E and 120E in Figure 4b), the solvent volume fraction in the swollen films was limited to below 0.35 $\left(\varphi_{s}<0.35\right)$. Even for the films exposed to the solvent for 120 min, $\varphi_{s}$ never exceeded 0.35, and subsequently achieved poor phase separation. Increasing the heating ramp to $2<r_{T}<3\;{}^{\circ}C/\min$ (samples 60C, 60D and 120C in Figure 4b) accelerated the solvent uptake to the range ($0.35<\varphi_{s}<0.5$) and achieved improved order in phase separation. The higher ramp regime, $r_{T}>4\;{}^{\circ}C/\min$ (samples 60A, 90A and 120A in Figure 4b), where the solvent volume fraction reached ($\varphi_{s}\geq 0.5)$ significantly enhanced the order in the microphase-separated films. As the effective interaction parameter (*χ_eff_*) between the blocks is inversely proportional to the solvent concentration in the swollen films, *χ_eff_* is decreased remarkably with a higher amount of solvent, which increases the chain mobility so to allow for a good phase separation with a high degree of order. According to our results, when the volume fraction of the solvent exceeds the polymer volume fraction in the swollen films, i.e., $\varphi_{s}\geq \varphi_{p}$, the films were phase separated with increased coherence length. The films annealed with a higher ramp during STVA appear to reach the threshold value of the solvent uptake $\left(\varphi_{s}\geq 0.5\right)$ faster than those films heated with a lower ramp. We postulate that a fast heating rate increases the vapour pressure of the THF on the surface of the film, and the difference between the pressure inside and outside of the film creates an inward osmotic pressure. Baruth et. al. [64] reported that an order–disorder state transition has been observed when PS-*b*-PLA films were exposed to THF solvent at a low vapour pressure and when $\varphi_{s}$ exceeds 0.58, whereas an increase in the correlation length was observed for the films annealed at a higher pressure. Sharp et al. [68] showed that when the relative pressure of the vapour increased in polylactide-co-glycolide polymer film, the amount of the solvent absorbed at equilibrium also increased. Additionally, quartz crystal microbalance analysis showed that increasing the vapour pressure led to an increase in the initial rate of the uptake but with a longer equilibrium time. In our study, we observed that the longer equilibrium time with a higher heating ramp allows the films to absorb a higher amount of solvent (samples 60A, 90A and 120A in Figure 4a). In contrast, when the temperature rises slowly, it leads to a lower solvent vapour activity (*a* = *P/P*_0_), thereby inhibiting $\varphi_{s}$ in reaching the threshold value. At a lower pressure ramp, the solvent reaches its equilibrium in a shorter time, before $\varphi_{s}$ reaches the threshold value.

### 3.3. Diffusion Kinetics

#### 3.3.1. Mass Uptake and Case II Diffusion

**Figure 5:**
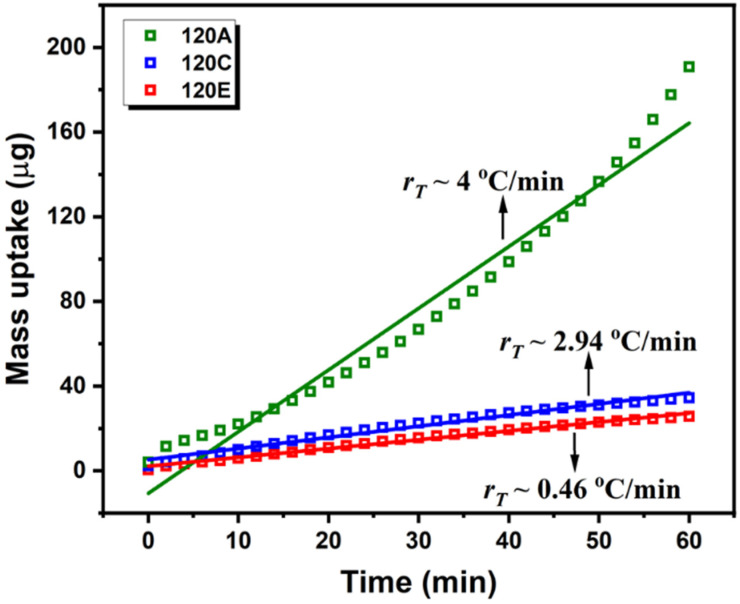
Evolution of mass uptake with respect to time for PS-*b*-PLA films exposed to THF solvent at different heating ramps in the oven. Linearly proportional mass uptake with time affirms Case II diffusion.

To study the kinetics of diffusion, the solvent mass uptake (*M*) was estimated from the solvent density (*ρ_s_*) and the increase in the film during the annealing. First, the swollen film thickness was extracted from $\varphi_{s}$ ($\varphi_{s}=1-\left(d_{0}/d\right)$, with $d_{0}$ being the initial dry film thickness, 217 nm). Then, M could be determined ($M=\rho_{s}V=\rho_{s\;}\left(d-d_{0}\right)*L*W$; *L* and *W* being the width and length of the substrate). The evolution of the mass uptake during the swelling is shown in Figure 5, illustrating the solvent diffusion kinetics for the PS-*b*-PLA film. Figure 5 clearly shows that the mass uptake increases as the solvent diffuses through the film and is linearly proportional to time, which is characteristic of Case II diffusion. At the beginning of the swelling process, the mass uptake is higher and deviates from linearity. The deviation is more pronounced at a higher heating ramp, which is expected due to the fast build-up of the vapour molecules readily diffusing through the surface of the film when the swelling starts. However, the swelling phenomenon is complex. More research is needed to explain the deviation from linearity in the initial stages by exposing the films at various diffusion rates. The penetrating solvent plasticises the polymer by reducing the glass transition temperature (*T_g_*), thereby allowing the solvent molecules to move easier into the polymer film in its glassy state. The solvent penetration rate is linear with respect to time for all the ramp regimes.

#### 3.3.2. Second Order Kinetics

**Figure 6:**
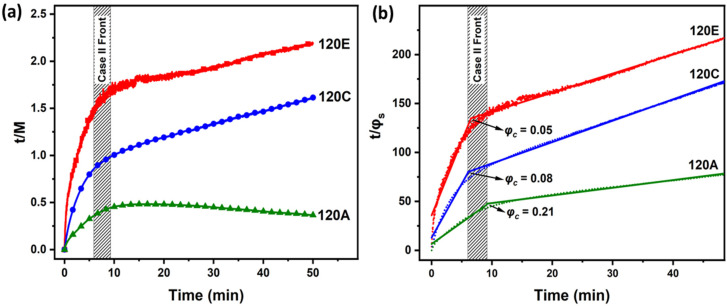
**Second order kinetics during swelling.** (**a**) Time over mass uptake (*t/M*) and (**b**) time over solvent volume fraction with respect to time. Films were annealed at different heating ramps, 120A ($r_{T}\sim 4\;{}^{\circ}C/\min$), 120C ($r_{T}\sim 2.94\;{}^{\circ}C/\min$ ) and 120E ($r_{T}\sim 0.46\;{}^{\circ}C/\min$ ). t/M is directly proportional to time, which is characteristic of second order kinetics. $t/\varphi_{s}$ vs. time showing two distinct stages in swelling order kinetics. Solid lines represent linear fits for the relevant curves.

The diffusion mechanism was further investigated by studying the kinetics of the swollen films. The linear equation for the second order kinetics for the one-dimensional thickness increase of the supported gelatine films proposed by Robinson [69], and used by Hans Schott [49] for the swollen polymer films, is shown in Equation (3).

(3) $$\frac{t}{M}=A+\frac{t}{M_{\infty }}$$

where *A* is a constant, *t* is time and *M* and $M_{\infty }$ are the mass uptakes at a given time and when the swelling reaches equilibrium, respectively. Figure 6a shows *t*/*M* versus time, plotted according to Equation (3), for the PS-*b*-PLA films exposed to THF at various heating ramps for 60 min. Irrespective of the ramp, all *t*/*M* profiles show the dual stage swelling order kinetics, as expected for Case II diffusion. In the early stage of swelling (<9 min, Figure 6a), the solvent is diffused through the film at a higher rate through the voids until the solvent uptake reaches a critical value $\left(\varphi_{c}\right)$ (Figure 6b). As the swelling progresses, the solvent diffusion is inhibited due to the stress relaxation of the polymer chains in a constrained area and where the Case II front is formed. In the propagation stage, the solvent moves through the film with a constant speed, thereby plasticising the polymer network. In recent studies [64,65], two solvent uptake regimes were observed before the target swelling level was reached while SVA was conducted in a custom-made rig apparatus, which is supportive to the present observations.

To extract the values of $\left(\varphi_{c}\right)$, we plotted $t/\varphi_{s}$ versus time, as shown in Figure 6b. Two distinct stages have been observed in the solvent fraction order kinetics, which is in good agreement with the observations from Figure 6a. In the early stage (i), the solvent molecules readily diffuse through the surface and $\varphi_{s}$ increases until a critical value ($\varphi_{c}$) is reached. An increase in the critical solvent volume fraction ($\varphi_{c}$) with an increased heating ramp has been observed. $\varphi_{c}$ values for sample 120E ($r_{T}\sim 0.4\;{}^{\circ}C/\min$), 120C ($r_{T}\sim 2.94\;{}^{\circ}C/\min$) and 120A ($r_{T}\sim 4\;{}^{\circ}C/\min$) are 0.05, 0.08 and 0.21, respectively. By achieving a larger $\varphi_{c}$ at an earlier stage of the swelling, induced by higher heating ramp, the area of plasticisation increases and the Case II front decelerates further [53,60]. The $\varphi_{c}$ values for 120C and 120E are similar, and the Case II front forms at 6 min, whereas for 120A, the Case II front decelerates and forms at 9 min. The higher solvent content in the early stage for sample 120A results in more plasticisation in the film by reducing the effective *T_g_*, which allows the solvent to penetrate further into the film and exceed the threshold $\varphi_{s}$ for microphase separation. At a higher heating ramp, the temperature of the substrate further reaches above the *T_g_* in shorter time intervals, which amplifies the chain mobility, along with a decrease in the effective Flory–Huggins interaction parameter (*χ_eff_*), as χ is inversely proportional to temperature. Similar to the early stage, the solvent diffusion rate is higher in the propagation stage for the films with the higher heating ramp in comparison to the lower ramp.

**Figure 7:**
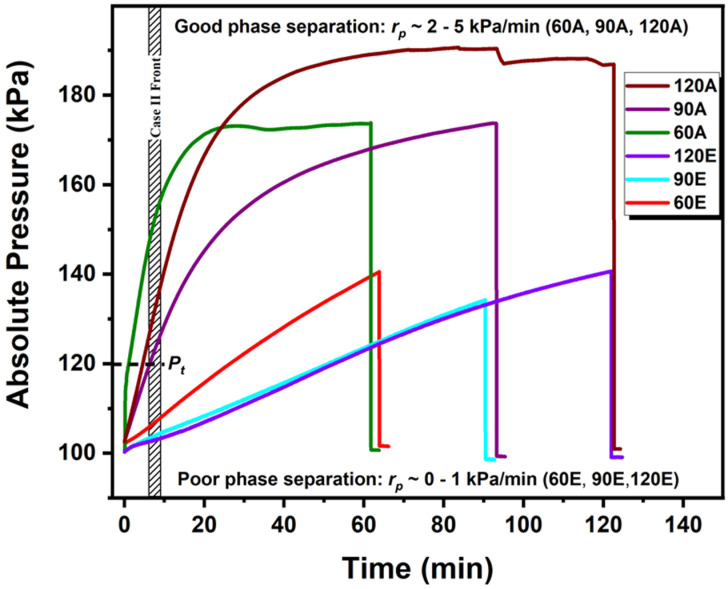
**Pressure ramp regimes.** Absolute vapour pressure profiles demonstrating the ramp regimes. Reaching threshold pressure (120 kPa) at early stage leads to phase separation with long-range order with higher vapour pressure ramp $\left(r_{P}>2\;\mathrm{kPa}/\min\right)$ and poor phase separation with lower pressure ramp $\left(r_{P}>1\;\mathrm{kPa}/\min\right)$.

The summary of the absolute vapour pressure profiles exhibiting good and poor microphase separation is demonstrated in Figure 7. The films annealed with a minimum pressure ramp of 2 kPa ($r_{P}>2\;\mathrm{kPa})$ promoted a good phase separation with a high translational order. The higher the ramp, the greater the long-range order. A pressure ramp of less than 1 kPa $\left(r_{P}<1\;\mathrm{kPa}\right)$ leads to poor phase separation. The temperature and the pressure ramp seem to be a more significant parameter for improving the order than the absolute value of the vapour pressure or the temperature only. The absolute pressure reaching the threshold pressure value of (120 kPa) in its early stage of swelling leads to good phase separation with a higher translational order. Reaching the threshold pressure later in the propagation stage, even at a longer annealing time, results in a poor phase separation. These results suggest that the minimum pressure ramp should be high ($r_{P}>2\;\mathrm{kPa}/\min)$ so to reach the threshold pressure in the early stage of the swelling. This is in agreement with earlier studies [64] that reported that the swelling rate in the second stage did not impact the final morphology of the film, which further emphasises the importance of the pressure ramp in the early stage. The experimental insights outlined here on the critical aspects of temperature and pressure ramps during SVA will aid in the further optimisation of the processing strategies in BCP systems and other polymeric thin film materials [63].

## 4. Conclusions

We investigated the swelling and diffusion kinetics of high-χ lamellar-forming PS-*b*-PLA films by exposing the thin films to THF vapour solvent at different temperature ramps, with the in situ monitoring of temperature and pressure. Identical films were annealed in a vapour environment at three different ramp regimes, low $(r_{T}<1{}^{\circ}C/\min$), medium $\left(2<r_{T}<3\;{}^{\circ}C/\min\right)$ and high $\left(r_{T}>4\;{}^{\circ}C/\min\right)$. As a result, the vapour pressure ramp ($r_{p}$) and the mass uptake were varied. The thin films exposed to the solvent vapour with lower ($r_{P}$) ($r_{P}<1\;\mathrm{kPa}/\min$), medium ($1<r_{P}<2\;\mathrm{kPa}/\min$) and higher ($r_{P}>2\;\mathrm{kPa}/\min$) regimes imparted poor, moderate and good phase separation, respectively. The results demonstrate the significance of a fast heating rate as an important parameter to achieve a highly ordered microphase-separated pattern. The swelling kinetics were demonstrated from the prospect of the evaluated solvent volume fraction and showed that a good phase separation is achieved only when ($\varphi_{s}\geq 0.5$) is reached. A longer annealing time with a slower heating and pressure rate neither improved the order in the pattern nor increased the coherence length significantly. The high osmotic pressure prompts the fast diffusion of the solvent species into the film, allowing the threshold volume fraction of the solvent $\left(\varphi_{s}\geq 0.5\right)$ to be reached. The calculated solvent mass uptake during the annealing was linearly proportional to time, indicating that solvent kinetics followed Case II diffusion. Further mass uptake analysis confirmed the second order kinetic mechanism during the swelling for the PS-*b*-PLA film. In summary, we observed that the essential parameters required to exhibit a good phase separation in our system are: (a) a minimum temperature ramp of $\left(r_{T}=4–5\;{}^{\circ}C/\min\right)$ and a pressure ramp of ($r_{P}=2–5\;\mathrm{kPa}$), and (b) a solvent volume fraction exceeding the polymer volume fraction, i.e., $\varphi_{s}\geq \varphi_{p}$. The experimental insights outlined here on the critical aspects of temperature and pressure ramps during STVA will aid in the further optimisation of the processing strategies in BCP systems and other polymeric thin film materials. Future work, which may involve the use of guiding templates, will benefit from this improved process window control.

## Figures and Tables

**Table 1:** List of the samples with relevant temperature and vapour pressure ramp annealed at three different regimes (slow, medium and fast heating) in the oven under THF solvent atmosphere for 60, 90 and 120 min.

Regimes	Slow Heating	Medium Heating	Fast Heating
$\mathrm{Heating}\;\mathrm{Rate}\;(r_{T})$ $\mathrm{Pressure}\;\mathrm{Rate}\;(r_{P})$	$r_{T}\;<\;1{}^{\circ}C/\min$ $r_{P}\;<\;1\;\mathrm{kPa}/\min$	$r_{T}\;=\;2–3\;{}^{\circ}C/\min$ $r_{P}\;=\;1–2\;\mathrm{kPa}/\min$	$r_{T}\;=\;4–5\;{}^{\circ}C/\min$ $r_{P}\;=\;1–2\;\mathrm{kPa}/\min$
Annealing time	Sample ID	Sample ID	Sample ID
60 min	60E, 60F	60C, 60D	60A
90 min	90E	90C	90A
120 min	120E	120C	120A

$r_{T}$ and $r_{P}$ are temperature ramp (°C/min) and pressure ramp (kPa/min), respectively.

## Data Availability

The data presented in this study are available on request from the corresponding author.

## Supplementary Materials

The following are available online at https://www.mdpi.com/article/10.3390/polym13234238/s1, Figure S1: Solvo-thermal vapour annealing (STVA) set up, Figure S2: Solvo-microwave annealing set up, Figure S3: Correlation profiles.
