# Supplementary material for: Defining Swelling Kinetics in Block Copolymer Thin Films: The Critical Role of Temperature and Vapour Pressure Ramp
Source: Polymers (Basel). 2021 Dec 3;13(23):4238. doi: 10.3390/polym13234238 (PMC8659708; doi:10.3390/polym13234238)
Supplement: Supplementary file 1 [file polymers-13-04238-s001.zip › polymers-1443619-SI.pdf]

Supplementary Information

Defining Swelling Kinetics in Block Copolymer Thin Films: The Critical Role of Temperature and Vapor Pressure Ramp

Sudhakara Neppalli,[a,c] Timothy W. Collins,[b] Zahra Gholamvand,[a,c] Cian Cummins,[d,e] Michael A. Morris,[a,b] and Parvaneh Mokarian-Tabari[a,c]*

(a) *School of Chemistry, The University of Dublin, Trinity College Dublin, Dublin, Ireland*

(b) *Department of Chemistry, University College Cork and Tyndall National Institute, Cork, Ireland.*

(c) *Advance Material and BioEngineering Research (AMBER) Centre and CRANN, Trinity College Dublin, Dublin, Ireland.*

(d) *CNRS, Univ. Bordeaux, Centre de Recherche Paul Pascal, UMR 5031, 115 Avenue Schweitzer, 33600 Pessac, France*

(e) *Univ. Bordeaux, CNRS, Bordeaux INP, LCPO, UMR 5629, F-33600 Pessac, France*

*Corresponding author: parvaneh.mokarian@tcd.ie

KEYWORDS: *Block copolymer, Kinetic mechanism, Phase separation, Solvent vapor Annealing, Heating rate, Pressure ramp*

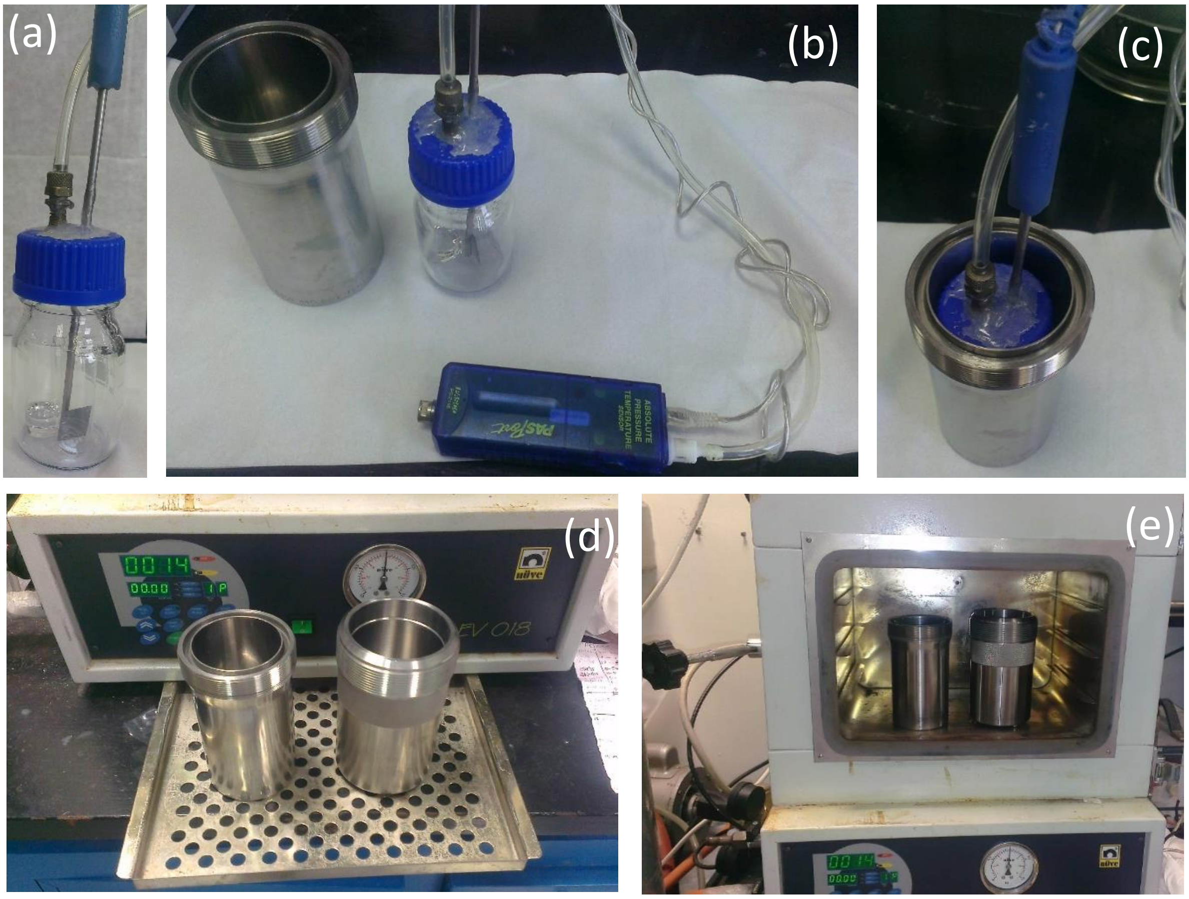

Figure S1. Solvo-Thermal Vapor Annealing (STVA) set up. (**a**) Air-tight borosilicate bell jar containing a 3 mL of THF solvent reservoir and silicon substrate coated with PS-*b*-PLA film with thickness of 217 ± 3 nm. A type K thermocouple was connected to the back of the substrate for *in-situ* monitoring substrate temperature. A pressure gauge and sensor were mounted on the top of the annealing jar to measure the pressure of the vapor inside the chamber *in-situ*. (**b**) Stainless steel chamber to locate the annealing jar and control the heating rate. (**c**) Top- down view of the annealing jar with connected temperature and pressure gauge inside the stainless steel chamber. (**d**) Stainless steel chamber was further placed on different thermally conducting material trays to create various heating ramp regimes. (**e**) The tray along with the annealing chamber was placed in the oven, where the target temperature was set at 55 °C, to perform the STVA experiments.

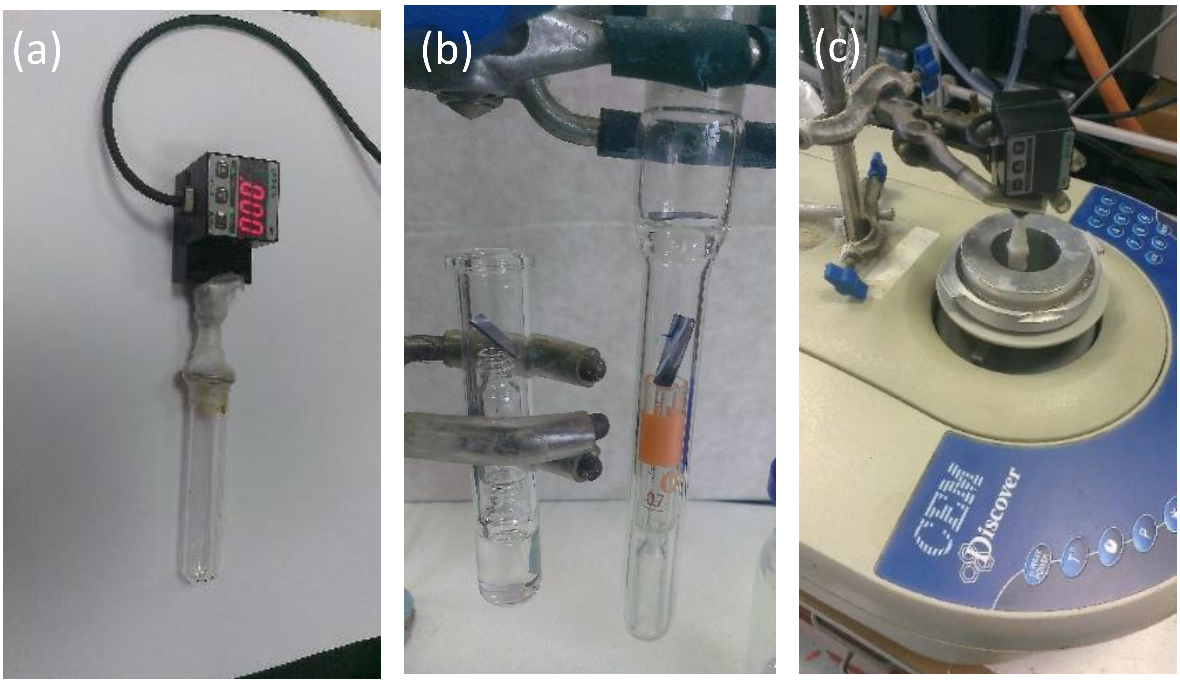

Figure S2. Solvo-Microwave Annealing set up. (**a**) a custom-made microwave tube with a pressure sensor assembled on top to allow monitoring of the vapor pressure during microwave annealing. (**b**) small jars containing THF, with the Si substrate in the microwave tube. (**c**) the glass tube with the pressure gauge is places in the microwave cavity to perform the solvo-microwave annealing experiments.

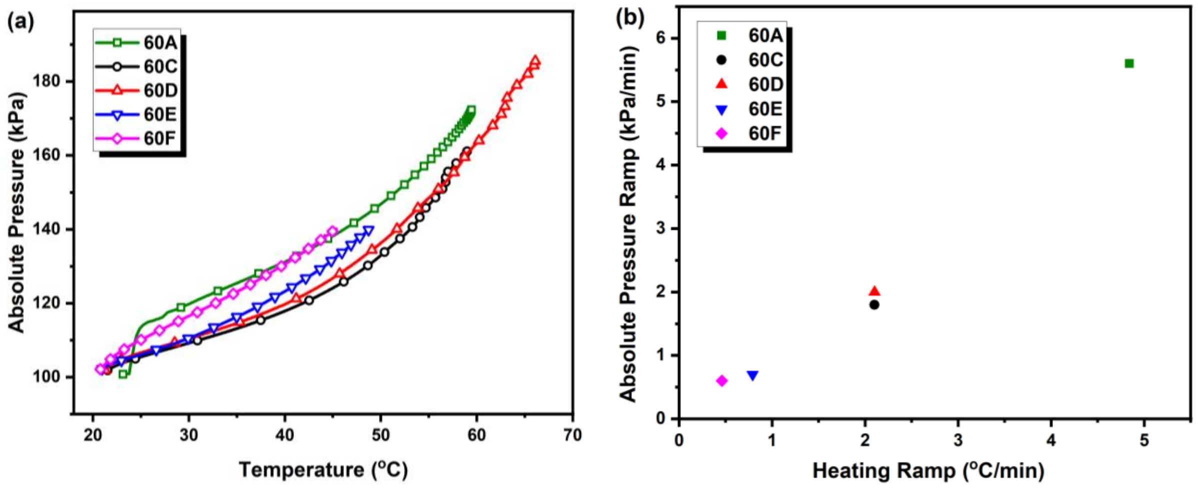

Figure S3. Correlation profiles (**a**) Absolute vapor pressure built up with respect to the temperature (**b**) Correlation between the absolute pressure ramp and heating ramp.
